# Supplementary material for: NF-κB Transcriptional Activity Indispensably Mediates Hypoxia–Reoxygenation Stress-Induced microRNA-210 Expression
Source: Int J Mol Sci. 2023 Apr 1;24(7):6618. doi: 10.3390/ijms24076618 (PMC10095479; doi:10.3390/ijms24076618)
Supplement: Supplementary file 1 [file ijms-24-06618-s001.zip › ijms-2295966-supplementary.pdf]

Supplementary Data and Materials

Homo sapiens chromosome 11, GRCh38.p14 Primary Assembly

NCBI Reference Sequence: NC_000011.10

>NC_000011.10:567589-568198 Homo sapiens chromosome 11, GRCh38.p14 Primary Assembly

Hybridization region 2 = NC_000011.10:567589-567663

CACGACCCGGTCCTGATTTTAACAGTAGACTTGAGAAGGCAGGCTCAGCAGACAGGCTGCTGGAGGATCC

ACCCGCTGTCTCCCGCAGCGGCCCCACTGAAGCGGCAGAAACACACAGAAGCACCAAATCTGTCACTCTC

NF-κB binding site 1 = NC_000011.10:567736-567747
-354 to -343 bp upstream of TSS

TTCCCG***TAGGAAGTCCCC***AGAGGTGGCCCTCCGGAGGATGGTCCTGTTGGCTGAAGATATGACCACACCT

GTGCCTGCCCAGGGCCACCTTCAGCTTGGCAACAGGAACTCCGCACACTGGTACCCTGGACACACAAGGA

p65 NF-κB binding site 2 = NC_000011.10:567873-567882
-216 to -207 bp upstream of TSS

AAAG***AAAGTTCCCT***CCAAGAGGGTATCTGGCCCAGCCTCAGCCCCACCAAGCGGAGGGCAGCAAAGCCCC

CGAATGATTTCGCTTACCCCCACCCGCCGTCCCTGCTGGCCAGACAGGCACACCCCGTCCATAGGGCACC

CCCTCCCCACGGTATCCAGCTGCCCTCGCGTCCCCGTGTGGCCGCTCGCCTGCCCGCCAGACCCGGACAC

Hybridization region 1 = NC_000011.10:568014-568088

TSS = NC_000011.10:568089

GGGGCCAGGAGGGTCGCGCTGCCCAGGCACAGATCAGCCGCTGTCACACGCACAGTGGGTCTGGGGCAGC

GCAGTGTGCGGTGGGCAGGGGCTGCCCTGCGCCTGGAGGCACTGCCGGGT

- miR-210 proximal promoter region (500 bp upstream of the TSS) NC_000011.10:567589-568088
- miR-210 coding region (110 bp downstream of the TSS) NC_000011.10:568089-568198

TSS: Transcription Start Site - NC_000011.10:568089

Supplementary Figure S1. *In-silico* analysis of the miR-210 proximal promoter (500 bp upstream of the TSS) harboring the NF-κB binding site, used to conceive the experimental approach and strategy to design the biotin-labeled miR-210 promoter *capture probe* and the *detection probe.*
TSS: transcription start site.

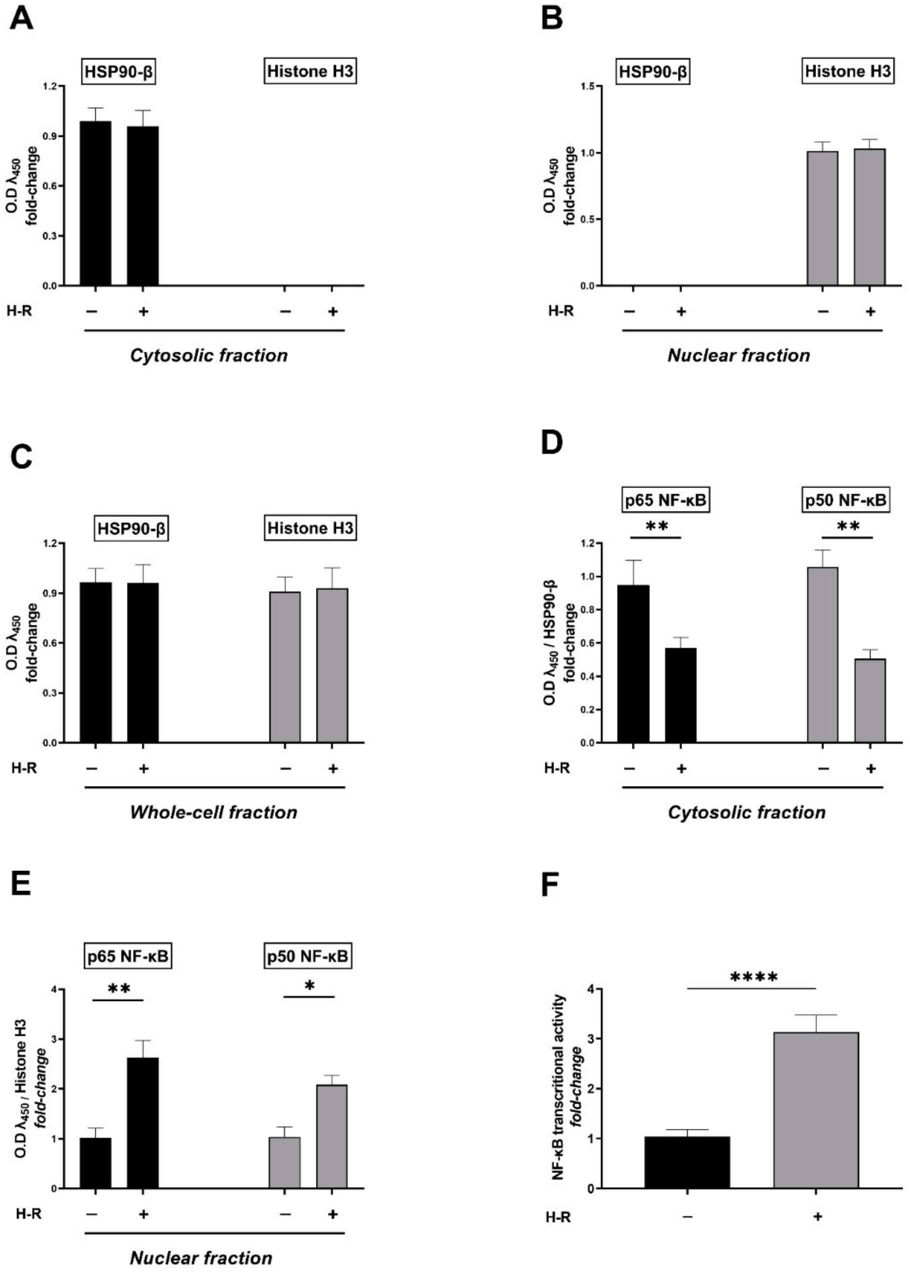

Supplementary Figure S2. Hypoxia-Reoxygenation (H-R) challenge evokes NF-κB activation. (A-C) Validity and integrity of the fractionated *cytosolic* and *nuclear* compartments subjected to quantitative ELISA immunoassays determining the abundance of p65 NF-κB and p50 NF-κB in the respective subcellular compartments. The integrity of the *cytosolic* fraction was validated by the presence of HSP90-β concomitant with the absence of Histone H3, while the integrity of the *nuclear* fraction was validated by the absence of HSP90-β concomitant with the presence of Histone H3. Quantitative sandwich ELISA immunoassays, determining the presence and relative abundance of HSP90-β and Histone H3 in the *cytosolic* fraction (A), *nuclear* fraction (B), as well as the whole-cell lysates (C), unequivocally demonstrate the fractional integrity and validity of the respective subcellular compartments. (D,E) Quantitative ELISA immunoassays determining the abundance of the p65 NF-κB and p50 NF-κB in the *cytosolic* fractions (D) and the *nuclear* fractions (E). (F) NF-κB transcriptional activity reporter assay determining the abundance of NF-κB-driven SEAP (Secreted Alkaline Phosphatase) expression levels in the conditioned media, as a surrogate of NF-κB transcriptional activity. All data is expressed as *Mean ± S.D fold-change* from three (3) technical replicates for each

of the four biological replicates belonging to each experimental group (n=4). *** p ≤ 0.05; ** p ≤ 0.01; **** p ≤ 0.0001; ns: not significant (p > 0.05).** S.D: standard deviation.

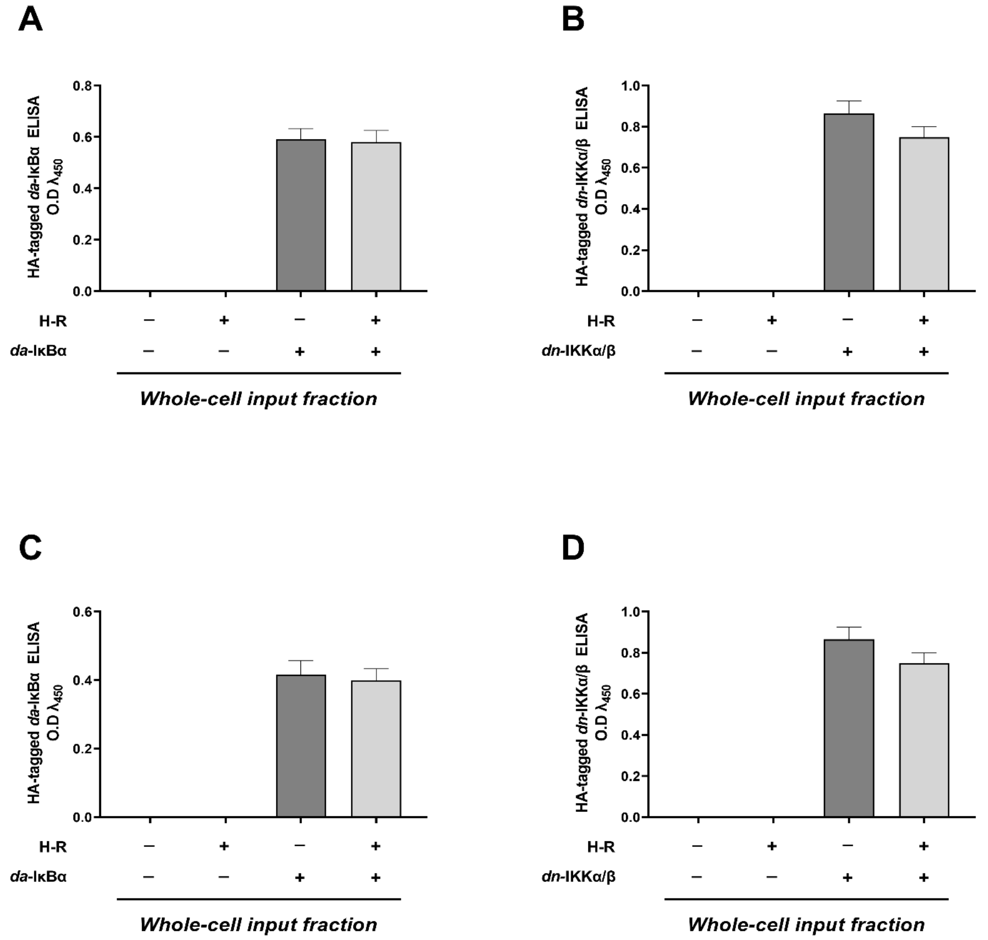

Supplementary Figure S3. Validation of ectopic expression of the HA-tagged *da*-IκBα mutant and the HA-tagged *dn*-IKKα/β mutants in native lysates subjected to miR-210 hybridization immunoassay as well as p65 ChIP-ELOHA analysis of the miR-210 promoter. (A-D) ELISA immunoassay executed against the HA-tag validating the ectopic expression of the HA-tagged *da*-IκBα mutant (A,C) and the HA-tagged *dn*-IKKα/β mutants (B,D) in the respective *native* lysates subjected to miR-210 hybridization immunoassay (A,B) as well as p65 ChIP-ELOHA analysis of the miR-210 promoter (C,D). Data is expressed as experimental blank-corrected absorbances (O.D) measured at λ_{450} (450 nm). Data is expressed as mean ± S.D from three technical replicates for each of the four biological replicates belonging to each experimental group (n = 4). O.D: optical density; S.D: standard deviation.

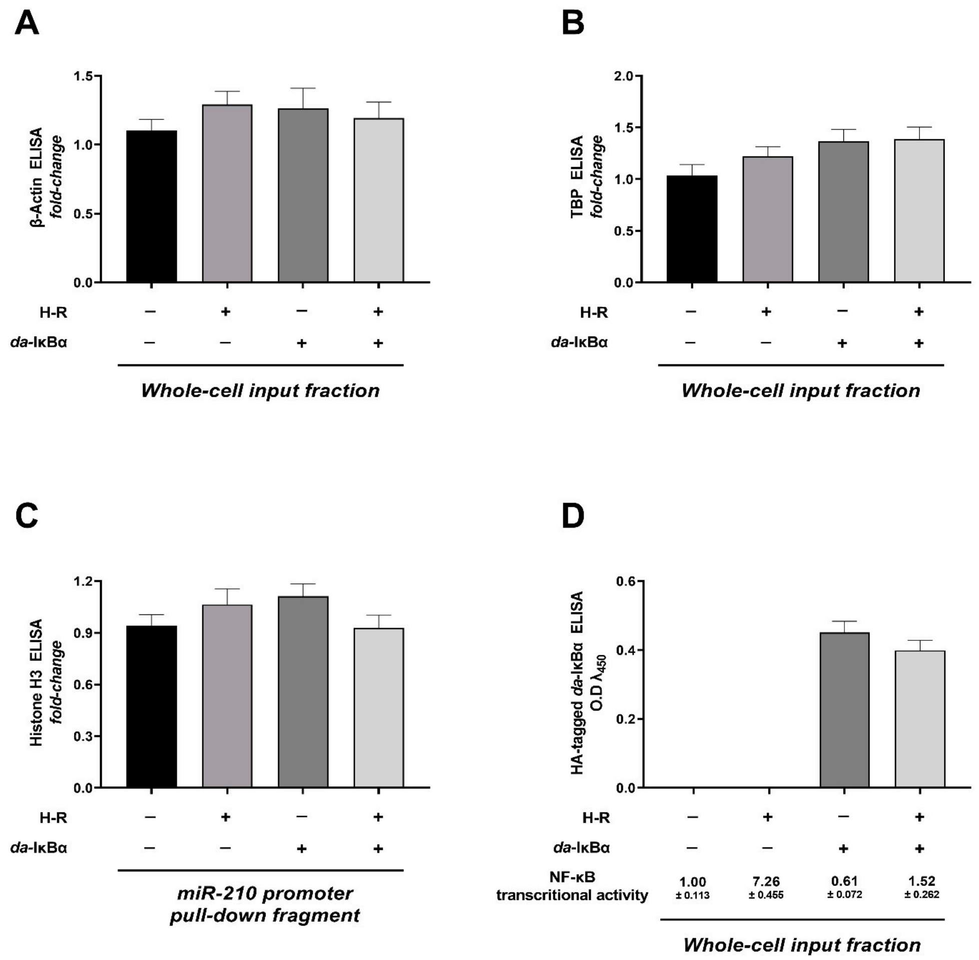

Supplementary Figure S4. Validation of equitable loading-inputs as well as equitable fractions of miR-210 promoter pull-down in the reverse cross-linked Reverse Chromatin Immunoprecipitation (R-ChIP) samples. **Scheme 210.** promoter bound NF-κB transcriptional coactivators. (p300 and CBP) and NF-κB transcriptional corepressors (SMRT and NCoR1). (A,B) Quantitative *sandwich.* ELISA immunoassays showing the equitable relative abundance of β-Actin (A) and the chromatin-associated TBP (B) in native cellular lysates subjected to the R-ChIP effectuated miR-210 promoter pull-down. (C) Quantitative *sandwich* ELISA immunoassay showing the equitable relative abundance of the chromatin-associated Histone H3 (C) in R-ChIP effectuated miR-210 promoter pull-down fragment. (D**)** ELISA immunoassay executed against the HA-tag validating the ectopic expression of the HA-tagged *da*-IκB*α* mutant. (D *bottom panel*) NF-κB transcriptional activity reporter assay determining the abundance of NF-κB-driven SEAP (Secreted Alkaline Phosphatase) expression levels in the conditioned media, as a surrogate of NF-κB transcriptional activity. Data from the NF-κB transcriptional activity reporter assay (D *bottom panel*) as well as the β-Actin (A), TBP (B), and Histone H3 (C) ELISA immunoassays, is expressed as *Mean ± S.D fold-change* from three (3) technical replicates for each of the four biological replicates belonging to each experimental group (n=4). Data from the HA-tag ELISA immunoassay is expressed as experimental blank-corrected absorbances (O.D) measured at λ_{450} (450 nm) depicted as mean ± S.D from three technical replicates for each of the four biological replicates belonging to each experimental group (n = 4). O.D: optical density; S.D: standard deviation.

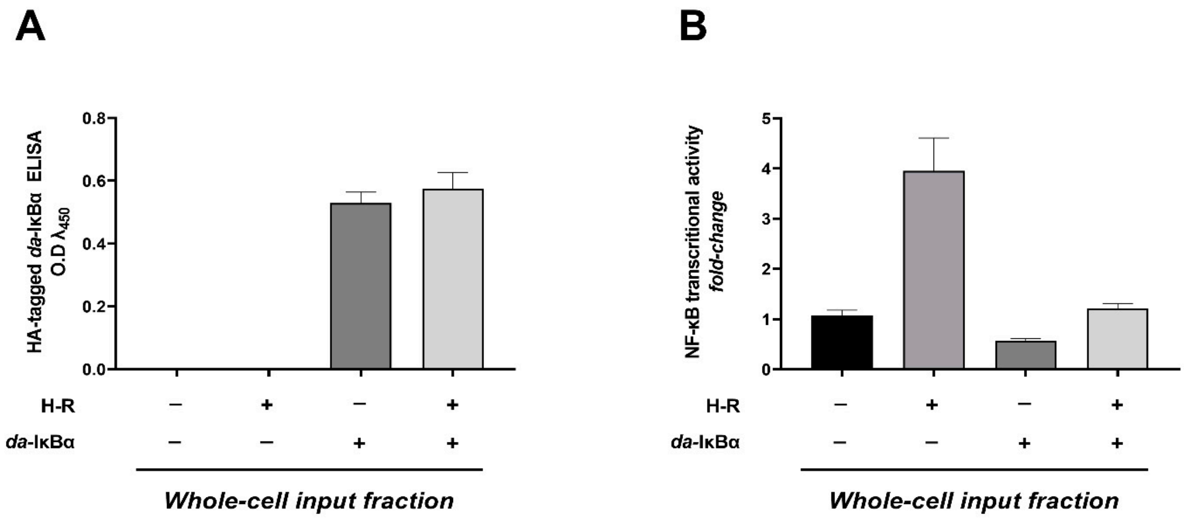

Supplementary Figure S5. Validation of ectopic expression of the HA-tagged *da*-IκBα mutant, as well as the determination of NF-κB transcriptional activity, in cellular inputs subjected to ChIP-ELOHA based RNAPII occupancy assay. (A) ELISA immunoassay executed against the HA-tag validating the ectopic expression of the HA-tagged *da*-IκBα mutant in cellular inputs subjected to ChIP-ELOHA based RNAPII occupancy assay. (B) NF-κB transcriptional activity reporter assay determining the abundance of NF-κB-driven SEAP (Secreted Alkaline Phosphatase) expression levels in the conditioned media, as a surrogate of NF-κB transcriptional activity, in cellular inputs subjected to ChIP-ELOHA based RNAPII occupancy assay. Data from the HA-tag ELISA immunoassay (A) is expressed as experimental blank-corrected absorbances (O.D) measured at λ_{450} (450 nm) depicted as mean ± S.D from three technical replicates for each of the four biological replicates belonging to each experimental group (n = 4). Data from the NF-κB transcriptional activity reporter assay (B) is expressed as *Mean ± S.D fold-change* from three (3) technical replicates for each of the four biological replicates belonging to each experimental group (n=4). O.D: optical density; S.D: standard deviation.

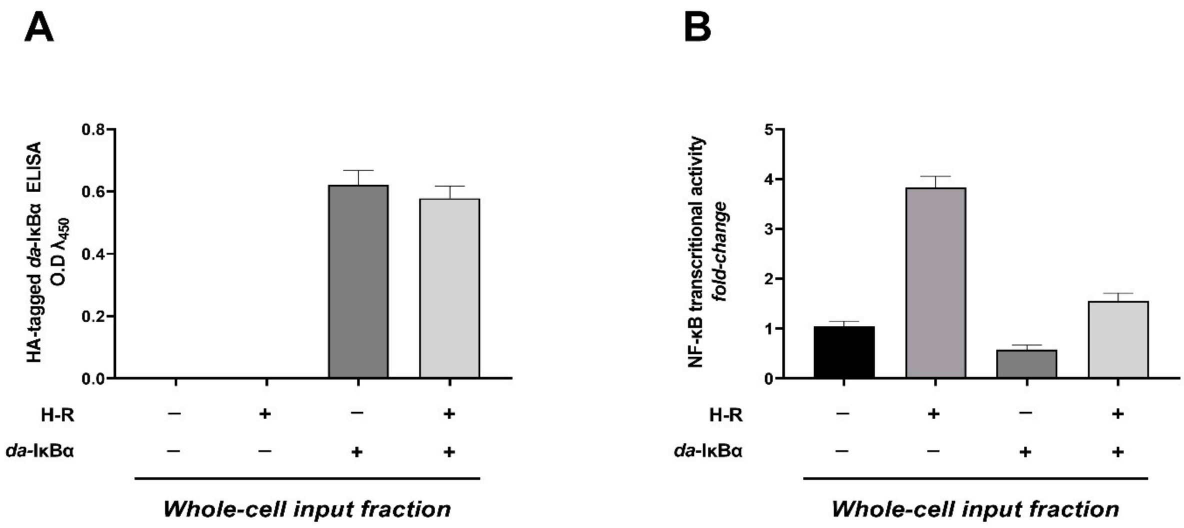

Supplementary Figure S6. Validation of ectopic expression of the HA-tagged *da*-IκBα mutant, as well as the determination of NF-κB transcriptional activity, in cellular inputs subjected to ChIP-ELOHA based Histone modification assays. (A) ELISA immunoassay executed against the HA-tag validating the ectopic expression of the HA-tagged *da*-IκB*α* mutant in cellular inputs subjected to ChIP-ELOHA based Histone modification assays. (B) NF-κB transcriptional activity reporter assay determining the abundance of NF-κB-driven SEAP (Secreted Alkaline Phosphatase) expression levels in the conditioned media, as a surrogate of NF-κB transcriptional activity, in cellular inputs subjected to ChIP-ELOHA based Histone modification assays. Data from the HA-tag ELISA immunoassay (A) is expressed as experimental blank-corrected absorbances (O.D) measured at λ_{450} (450 nm) depicted as mean ± S.D from three technical replicates for each of the four biological replicates belonging to each experimental group (n = 4). Data from the NF-κB transcriptional activity reporter assay (B) is expressed as *Mean ± S.D fold-change* from three (3) technical replicates for each of the four biological replicates belonging to each experimental group (n=4). O.D: optical density; S.D: standard deviation. .

REVERSE COMPLEMENT of the miR-210 proximal promoter region

Homo sapiens chromosome 11, GRCh38.p14 Primary Assembly

NCBI Reference Sequence: NC_000011.10

>NC_000011.10:c568088-567589 Homo sapiens chromosome 11, GRCh38.p14 Primary Assembly

Hybridization probe 1 = NC_000011.10:c568088-568014

TCCTGGCCCCGTGTCCGGGTCTGGCGGGCAGGCGAGCGGCCACACGGGGACGCGAGGGCAGCTGGATACC

GTGGGGAGGGGGTGCCCTATGGACGGGGTGTGCCTGTCTGGCCAGCAGGGACGGCGGGTGGGGGTAAGCG

AAATCATTCGGGGGCTTTGCTGCCCTCCGCTTGGTGGGGCTGAGGCTGGGCCAGATACCCTCTTGGAGGG

AACTTTCTTTTCCTTGTGTGTCCAGGGTACCAGTGTGCGGAGTTCCTGTTGCCAAGCTGAAGGTGGCCCT

GGGCAGGCACAGGTGTGGTCATATCTTCAGCCAACAGGACCATCCTCCGGAGGGCCACCTCTGGGGACTT

CCTACGGGAAGAGAGTGACAGATTTGGTGCTTCTGTGTGTTTCTGCCGCTTCAGTGGGGCCGCTGCGGGA

Hybridization probe 2 = NC_000011.10:c567663-567589

GACAGCGGGTGGATCCTCCAGCAGCCTGTCTGCTGAGCCTGCCTTCTCAAGTCTACTGTTAAAATCAGGA

CCGGGTCGTG

REVERSE COMPLEMENT of the miR-210 proximal promoter region (500 bp upstream of the TSS [Transcription Start Site])

NC_000011.10:c568088-567589

Hybridization probe 1
TCCTGGCCCCGTGTCCGGGTCTGGCGGGCAGGCGAGCGGCCACACGGG
GACGCGAGGGCAGCTGGATACCGTGGG

Hybridization probe 2
CGGGTGGATCCTCCAGCAGCCTGTCTGCTGAGCCTGCCTTCTCAAGTCT
ACTGTTAAAATCAGGACCGGGTCGTG

Supplementary Figure S7. The *reverse complement* sequence of the miR-210 proximal promoter region depicting the oligonucleotide sequences utilized to design the *capture probe* and the *detection probe* the miR-210 promoter pull-down and the ELOHA analysis. ELOHA: enzyme-linked oligonucleotide hybridization assay.

Supplementary Table S1. Composition of the *hypoxia medium.*

Component	500 mL	Final concentration	Source (Notation)
DMEM, No Glucose	464.45 mL	93% v/v	1
Creatine	131.2 mg	5 mM	2
D-(+)-Glucose Solution 2.5 M, 450 g/L	0.55 mL	2.75 mM	3
Glutamine 200 mM	5 mL	2 mM	4
HEPES 1M	5 mL	10 mM	5
L-Carnitine, 200 mM	5 mL	2 mM	6
Non-essential Amino Acids, 100x	5 mL	N/A*	7
Sodium Pyruvate 100 mM	5 mL	1 mM	8
Taurine 500 mM	5 mL	5 mM	9
Linoleic Acid-Oleic Acid-Albumin, 100x	5 mL	N/A*	10

Supplementary Table S1: Notation legend

[1] Thermo Fisher Scientific, Oslo, Norway, Catalogue # 11966025

[2] Sigma Aldrich / Merck Millipore / Merck Life Science, Darmstadt, Germany, Catalogue # C3630-100G

[3] Sigma Aldrich / Merck Millipore / Merck Life Science, Oslo, Norway, Catalogue # G8769

[4] Thermo Fisher Scientific, Oslo, Norway, Catalogue # A2916801

[5] Sigma Aldrich / Merck Millipore / Merck Life Science, Darmstadt, Germany, Catalogue # H4034-500G

[6] Sigma Aldrich / Merck Millipore / Merck Life Science, Darmstadt, Germany, Catalogue # C0283-25G

[7] Thermo Fisher Scientific, Oslo, Norway, Catalogue # 11140035

[8] Thermo Fisher Scientific, Oslo, Norway, Catalogue # 11360070

[9] Sigma Aldrich / Merck Millipore / Merck Life Science, Darmstadt, Germany, Catalogue # T8691-100G

[10] Sigma Aldrich / Merck Millipore / Merck Life Science, Darmstadt, Germany, Catalogue # L9655-5ML

* N/A - Not Applicable
